# The prognostic role of the cancer stem cell marker CD44 in ovarian cancer: a meta-analysis

**DOI:** 10.1186/s12935-016-0376-4

**Published:** 2017-01-05

**Authors:** Jiaying Lin, Ding Ding

**Affiliations:** 1 Department of Assisted Reproduction, Shanghai Ninth People’s Hospital, School of Medicine, Shanghai Jiao Tong University, 639 Zhizaoju Road, Shanghai, 200011 China; 2 Department of Gynecology, Obstetrics and Gynecology Hospital, Fudan University, 419 Fangxie Road, Shanghai, 200011 China

**Keywords:** Ovarian cancer, Cancer stem cells, CD44, Prognosis

## Abstract

**Background:**

CD44 has recently been reported as a cancer stem cell marker in ovarian cancer. However, the clinicopathological and prognostic value of this marker in ovarian cancer remains controversial; Here, we aimed to investigate the correlation between CD44 expression and the clinicopathological features or survival of ovarian cancer patients.

**Methods:**

An extensive literature search in the PubMed, EMBASE, and Wanfang databases (up to June 1, 2016) was conducted to identify studies that assessed the clinical or prognostic significance of CD44 expression in ovarian cancer. A meta-analysis was then performed to clarify the association between CD44 expression and clinical outcomes of ovarian cancer patients.

**Results:**

A total of 18 publications consisting of 2161 patients were included for this meta-analysis. Our data reveal that CD44-positive expression in ovarian cancers were significantly associated with a high TMN stage (pooled OR = 2.11, 95% CI 1.26–3.53, P = 0.004) and poor 5-year overall survival (RR = 1.42, 95% CI 1.01–2.00, P = 0.05). However, CD44 expression was not associated with tumor grade, lymphatic metastasis, age of the patients, residual tumor size, response to chemotherapy, or ascites volume (P > 0.05).

**Conclusion:**

Detection of CD44 may be an effective tool for pathological diagnosis and prognostic prediction of ovarian cancer patients in clinical applications.

## Background

Ovarian cancer is the leading cause of death from gynecologic malignancy [1]. Although most ovarian cancer patients initially respond very well to platinum-based chemotherapy, the vast majority of patients will ultimately develop cancer recurrence and succumb to chemo-resistant disease [1].

CSCs represent a subpopulation of cancer cells that possess tumor initiation and self-renewal capacity and have been implicated in driving tumor growth, metastasis, and relapse following therapy in a wide variety of human cancers such as breast and ovarian cancers [2, 3]. In recent years, CSCs have also been found to contribute to the poor clinical outcome of ovarian cancer patients [4]. Furthermore, it has been suggested that CSC markers, such as CD44, ALDH1 and CD133, may serve as valuable prognostic indicators for ovarian cancer [4, 5]. Among these CSC markers, CD44 is the most frequently reported in ovarian cancer. Several studies have demonstrated that CD44 can be used to isolate cancer cells with stem cell-like and cancer-initiating properties from other populations of cancer cells [6–8].

CD44, which consists of four functional domains, plays important roles in cell–matrix adhesion, signal transduction, cytoskeletal rearrangement, and cell migration [9]. Intriguingly, the proximal extracellular domain is the site for alternative splicing of the CD44 mRNA that results in various isoforms of CD44. The CD44 standard form (CD44s) and CD44 variants (CD44v) participate in cell–cell and cell–matrix interactions, cell migration, lymphocytehoming, and tumorigenesis. CD44v6 has particularly gained a lot of attention in recent years, since the expression of this variant has been found to be upregulated in a variety of epithelial malignancies, such as head and neck, colon, endometrium and ovarian cancers. CD44v6 may be implicated in the activation of PI3K/Akt and MAPK pathways, which can then inhibit apoptosis and promote invasion and metastasis of cancer cells [10–14].

Although the association between the expression of CD44 or its isoforms and the survival of patients with ovarian cancer has been well investigated, the prognostic values of CD44, CD44s, and CD44v6 in predicting the survival of patients with ovarian cancer remains controversial [15–19]. In the present study, we performed a systematic review and meta-analysis of the published literature to examine the association of the expression of CD44 or its isoforms with the clinicopathological features and the prognosis of ovarian cancer patients. These findings may help to uncover valuable marker that may enable the prognostic stratification of ovarian cancer patients and guide the management of these patients in the future.

## Methods

### Search strategy

The electronic databases, including Pubmed, Embase, and Wanfang, were searched for studies that investigated the correlation of the CD44 expression and clinicopathological parameters and prognosis in ovarian cancer patients. The literature search was updated until June 1, 2016. The search terms were used as follows: “CD44”, “ovarian neoplasms” or “ovary neoplasms” or “ovarian cancer” or “cancer of ovary”. Review articles and the citations from all the retrieved reports were further manually reviewed to identify other relevant publications. The titles and abstracts of identified reports were examined to exclude any irrelevant publications. The full-text of the remaining articles were further inspected to determine whether they reported the correlation of the expression of CD44 or its isoforms and the clinicopathological features and prognosis of ovarian cancer patients.

### Inclusion and exclusion criteria

The studies included for this meta-analysis met the following criteria: (1) Definitive diagnosis of ovarian cancer was made on the basis of histopathological findings; (2) Studies that examined the protein expression of CD44, CD44s, or CD44v6 in ovarian tissues instead of that in the serum or any other types of specimen; (3) studies that investigated the correlation of the expression of CD44, CD44s, or CD44v6 with clinicopathological features; (4) studies that reported the association of the expression of CD44 or its isoforms with overall survival (OS) of ovarian cancer patients. There was no limitation on the minimum number of patients in each study. When there were multiple articles by the same group based on similar patient populations, only the largest or the most recent article was included. The exclusion criteria for this meta-analysis include: (a) Studies were not associated with the topic of interest; (b) researchers did not make the definitive diagnosis based on histopathologic findings or they did not carried out clinical and imaging follow-up for at least 6 months; (c) studies associated with other diseases (d); non-original articles; (e) data could not be extracted; and (f) duplicate data from the same or similar patient population.

### Data extraction

The following information was extracted from the retrieved full-text papers: lead author, country of the patients, ethnicity, publication year, time of sample collection, the pathological stages of tumors, number of patients, types of research techniques, the ages of the patients, and the choice of cut-off scores for the definition of positive staining or staining intensity. The included studies can be divided into two major groups based on the research objectives of these studies. One group evaluated the correlation between the expression of CD44, CD44s, or CD44v6 and the clinicopathological parameters, including TMN stage, tumor grade, lymphatic metastasis, age of the patients, residual tumor size, responses to chemotherapy, and ascites volume. The other group investigated the association between the expression of CD44, CD44s, or CD44v6 and the OS or disease-free survival (DFS).

### Statistical analysis

The meta-analysis was performed as previously described [20]. Odds ratios (ORs) with 95% confidence interval (CI) were employed to evaluate the association between the expression of CD44, CD44s, or CD44v6 and the clinicopathological features for the patients with ovarian cancer, including TMN stage, tumor grade, lymphatic metastasis, age of the patients, residual tumor size, responses to chemotherapy, and ascites volume. By contrast, the risk ratio (RR) was used for assessing the correlation of the expression of CD44, CD44s, or CD44v6 and the OS or DFS. If RRs were not reported directly in the published articles, the data from those papers were then used to calculate the RR according to the methods described by Parmar et al. [21]. Heterogeneity across studies was evaluated with the Q test and P values. ORs and RRs were calculated using a random-effects model when the P value was less than 0.05. Otherwise, a fixed-effects model was applied. The Begg’s funnel plot and Egger’s test were used to assess publication bias. All statistical analyses were carried out using Review manager software (Revman version 5.3.5.). The difference will be considered statistically significant when two-sided P values are less than 0.05.

## Results

### Characteristics of the included studies

**Fig. 1:**
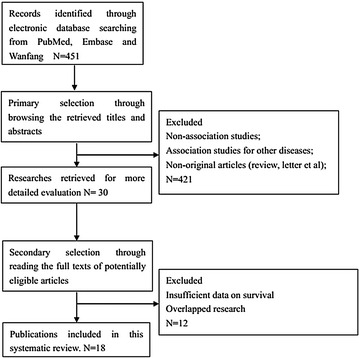
The flow diagram of article selection

A total of 451 articles were originally identified for the meta-analysis after searching the electronic databases PubMed, Embase, and Wanfang. 421 reports were excluded after closely reviewing the titles and abstracts. Eventually, the extensive review of the full-text articles yielded a total of 18 studies consisting of 2161 patients that met the inclusion criteria for this meta-analysis [15–19, 22–34] (Fig. 1). The main characteristics of the eligible studies are summarized in Table 1. Among the included studies, 17 articles assessed the correlation between the expression of CD44 or its isoforms and the clinicopathological features of ovarian cancer, whereas the association of CD44 expression with OS or DFS was examined using the Kaplan–Meier method in 12 of these studies.

**Table 1:** Characteristics of the included studies

Study	Patient’s country	Ethnicity	Year	Time of collection	Pathological stage	Number of patients	Age in years	Follow-up months	Cut-off for CD44 positive (% staining)	Survival analysis	Subtypes of the CD44 family	Method
Ross et al.	USA	Caucasian	2001	1991–1995	I–IV	101	30–85	70	>10	OS	CD44s	IHC
Cho et al.	Korea	Asian	2006	ND	I–III	158	ND	112	>50	OS	CD44s	IHC
Zhang et al.	China	Asian	2013	1990–2007	I–IV	483	22–89	120	>25	OS; DFS	CD44s	IHC
Zhong et al.	China	Asian	2001	1993–1996	I–IV	55	ND	ND	>10	ND	CD44v6	IHC
Rodríguez et al.	USA	Caucasian	2003	1990–1996	I–IV	142	ND	50	>50	OS; DFS	CD44v6	IHC
Hong et al.	Korea	Asian	2006	1997–2004	I–IV	65	25–74	ND	>10	ND	CD44v6	IHC
Xu et al.	China	Asian	2009	2005–2008	I–IV	63	26–80	ND	>5	ND	CD44v6	IHC
Shi et al.	China	Asian	2013	2010–2011	I–III	45	ND	ND	>25	ND	CD44v6	IHC
Tjhay et al.	Japen	Asian	2015	2002–2012	ND	59	37–82	140	>10	OS	CD44v6	IHC
Sillanpaa et al.	Finland	Caucasian	2003	1976–1992	I–IV	445	ND	237	>10	OS; DFS	CD44	IHC
Chen et al.	China	Asian	2009	2001–2007	I–IV	120	40–70	56	>25	DFS	CD44	IHC
Jiang et al.	China	Asian	2010	2007–2008	I–IV	33	33–74	ND	>50	ND	CD44	IHC
Gao et al.	USA	Caucasian	2015	ND	I–IV	26	ND	150	>25	OS; DFS	CD44	IHC
Steffensen et al.	Denmark	Caucasian	2011	ND	I–IV	109	32–79	40	>20	ND	CD44	IHC
Liu et al.	USA	Caucasian	2012	2006–2007	I–IV	33	44–86	60	>5	OS	CD44	IHC
Li et al.	China	Asian	2012	2007–2008	I–IV	46	ND	ND	>10	ND	CD44	IHC
Wang et al.	China	Asian	2015	2006–2012	I–IV	86	29–73	94	>50	OS	CD44	IHC
Zhu et al.	USA	Caucasian	2015	2006–2010	I–IV	92	24–78	84	>25	OS	CD44	IHC

*IHC* immunohistochemistry, *OS* overall survival, *DFS* disease-free survival, *ND* not document

### The correlation of CD44v6 expression with clinicopathological parameters

**Fig. 2:**
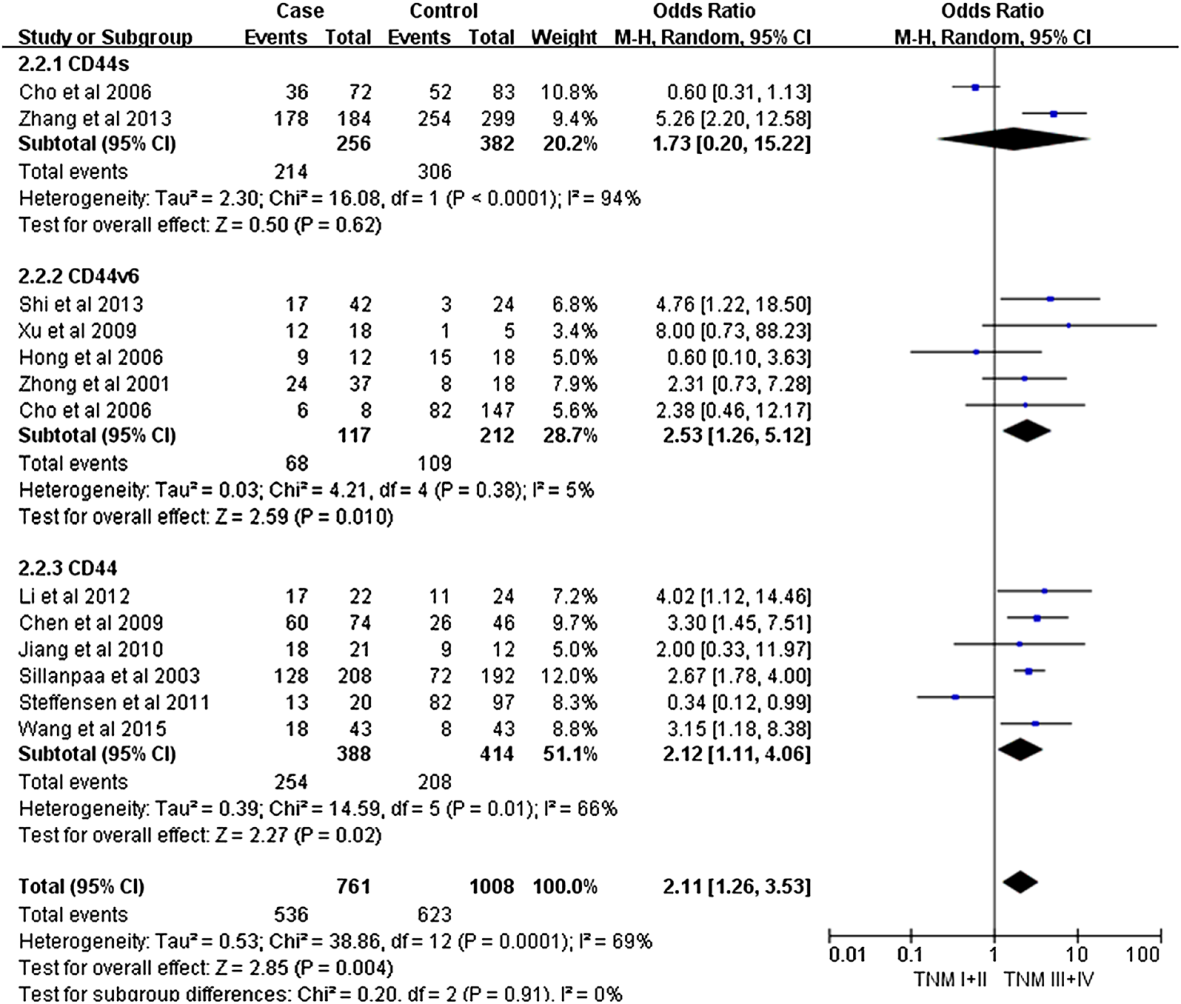
Forest plot depiction of CD44 expression and TMN stage

**Fig. 3:**
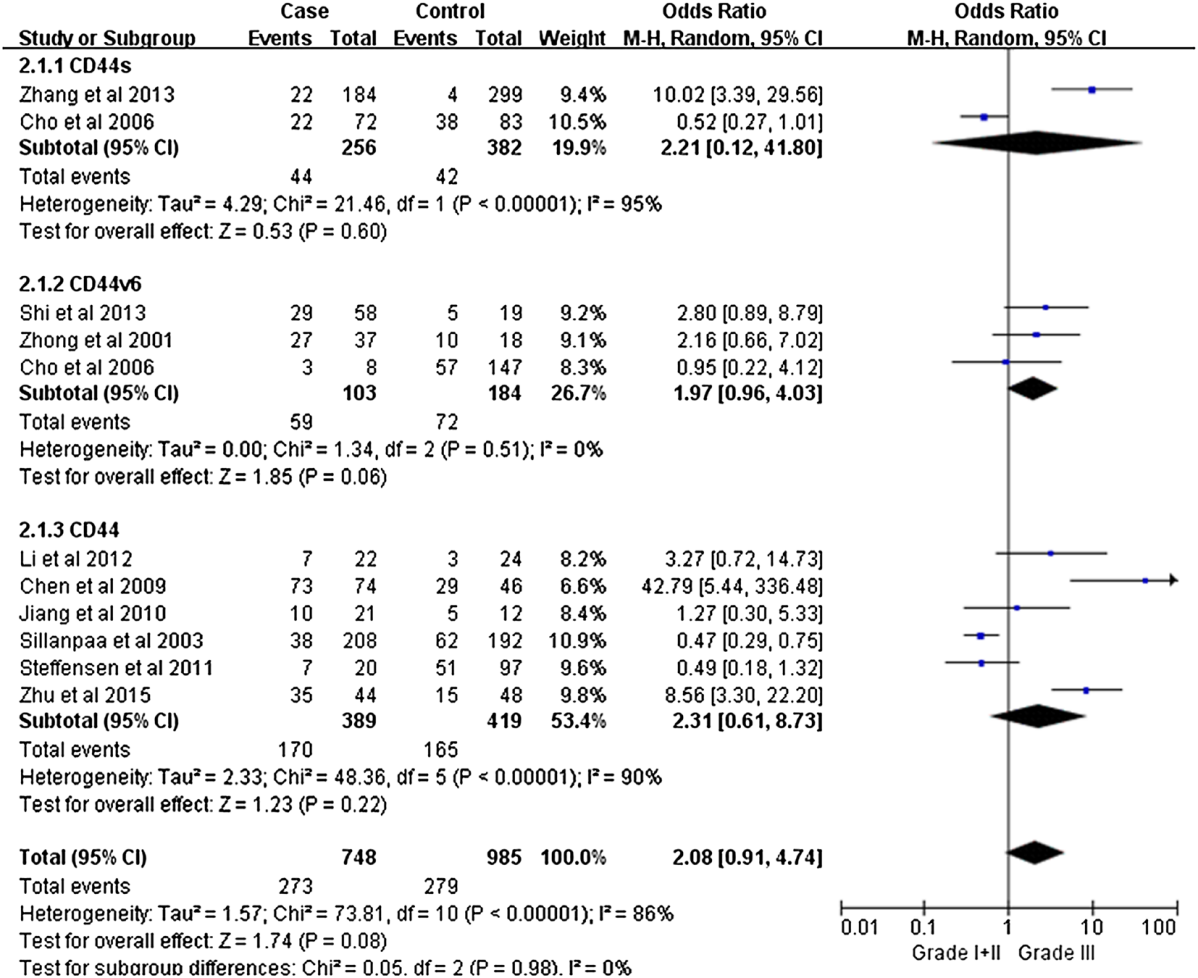
Forest plot depiction of CD44 expression and tumor grade

**Fig. 4:**
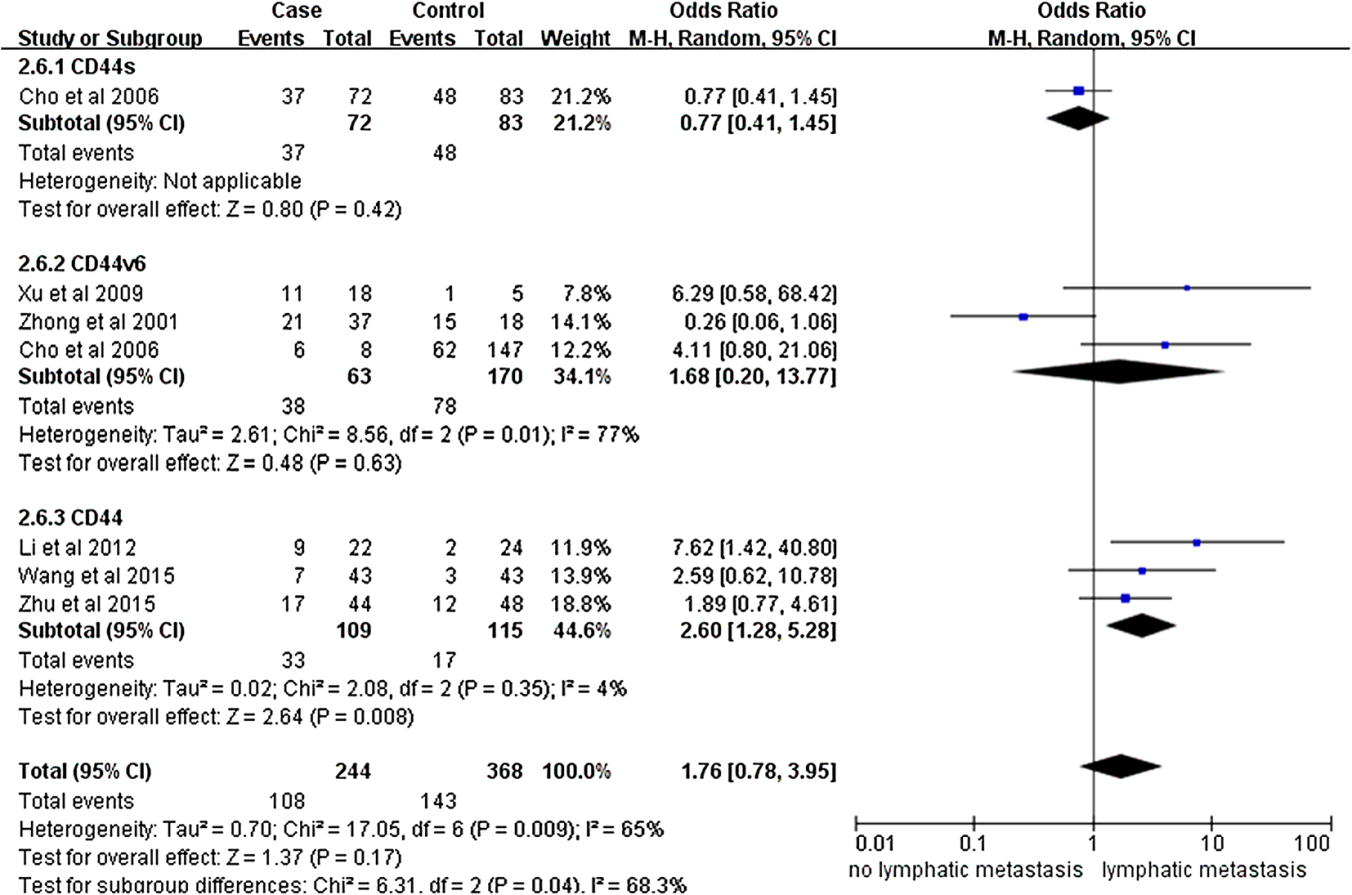
Forest plot depiction of CD44 expression and lymphatic metastasis

**Fig. 5:**
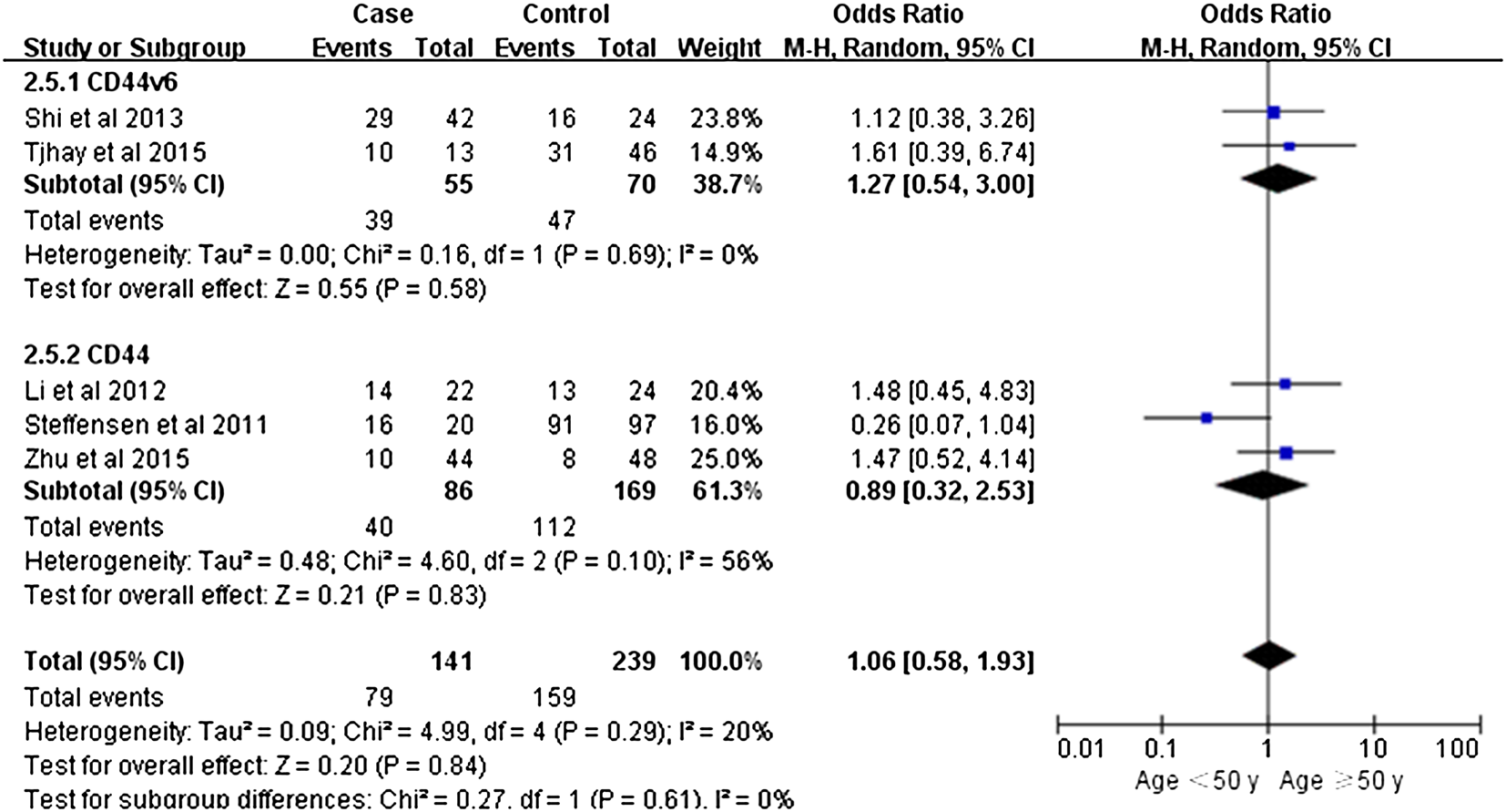
Forest plot depiction of CD44 expression and age of the patients

**Fig. 6:**
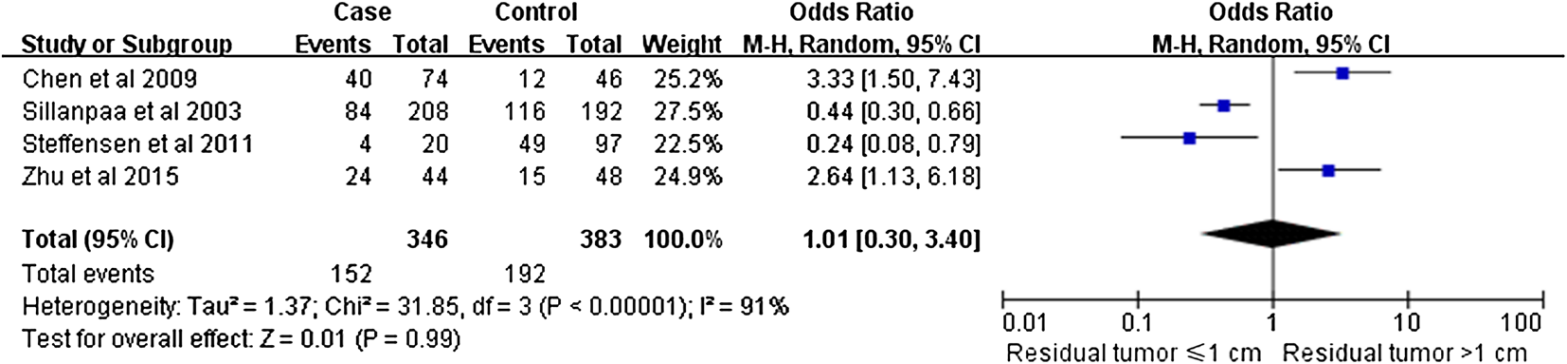
Forest plot depiction of CD44 expression and residual tumor size

**Fig. 7:**
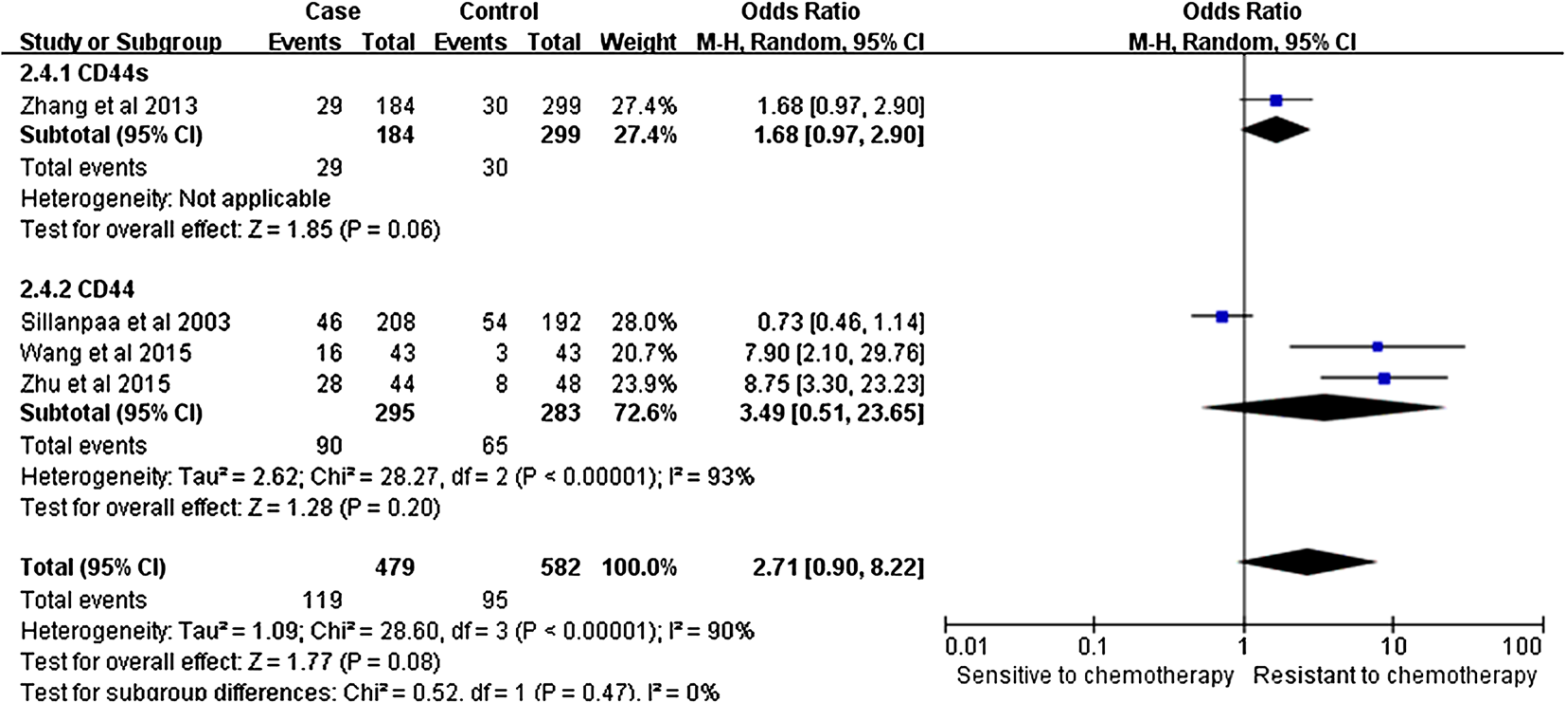
Forest plot depiction of CD44 expression and response to chemotherapy

**Fig. 8:**
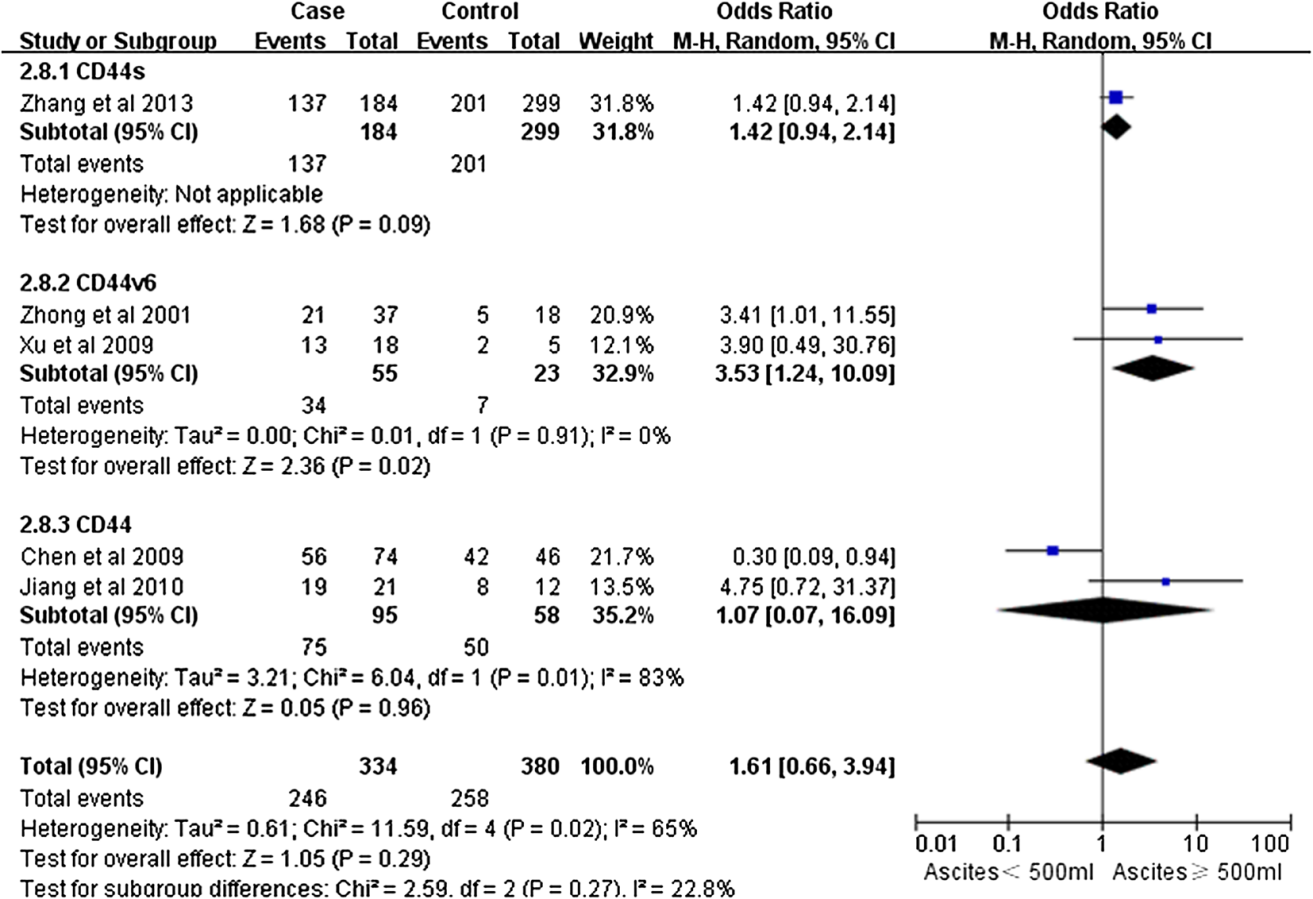
Forest plot depiction of CD44 expression and ascites volume l(G)

Notably, our analysis based on the random-effect model reveals that the expression of CD44v6 in ovarian cancers is significantly associated with a high TMN stage (pooled OR = 2.11, 95% CI 1.26–3.53, P = 0.004) (Fig. 2). However, there is no significant correlation between CD44 expression and tumor grade (pooled OR = 2.08, 95% CI 0.91–4.74, P = 0.08, random-effect model) (Fig. 3), lymphatic metastasis (pooled OR = 1.76, 95% CI 0.78–3.95, P = 0.17, random-effect model) (Fig. 4), age of the patients (pooled OR = 1.60, 95% CI 0.58–1.93, P = 0.84, fixed-effect model) (Fig. 5), residual tumor size (pooled OR = 1.01, 95% CI 0.30–3.40, P = 0.99, random-effect model) (Fig. 6), response to chemotherapy (pooled OR = 2.71, 95% CI 0.90–8.22, P = 0.08, random-effect model) (Fig. 7), or ascites volume (pooled OR = 1.61, 95% CI 0.66–3.94, P = 0.29, random-effect model) (Fig. 8).

### The correlation between CD44 expression and the prognosis of ovarian cancer

**Fig. 9:**
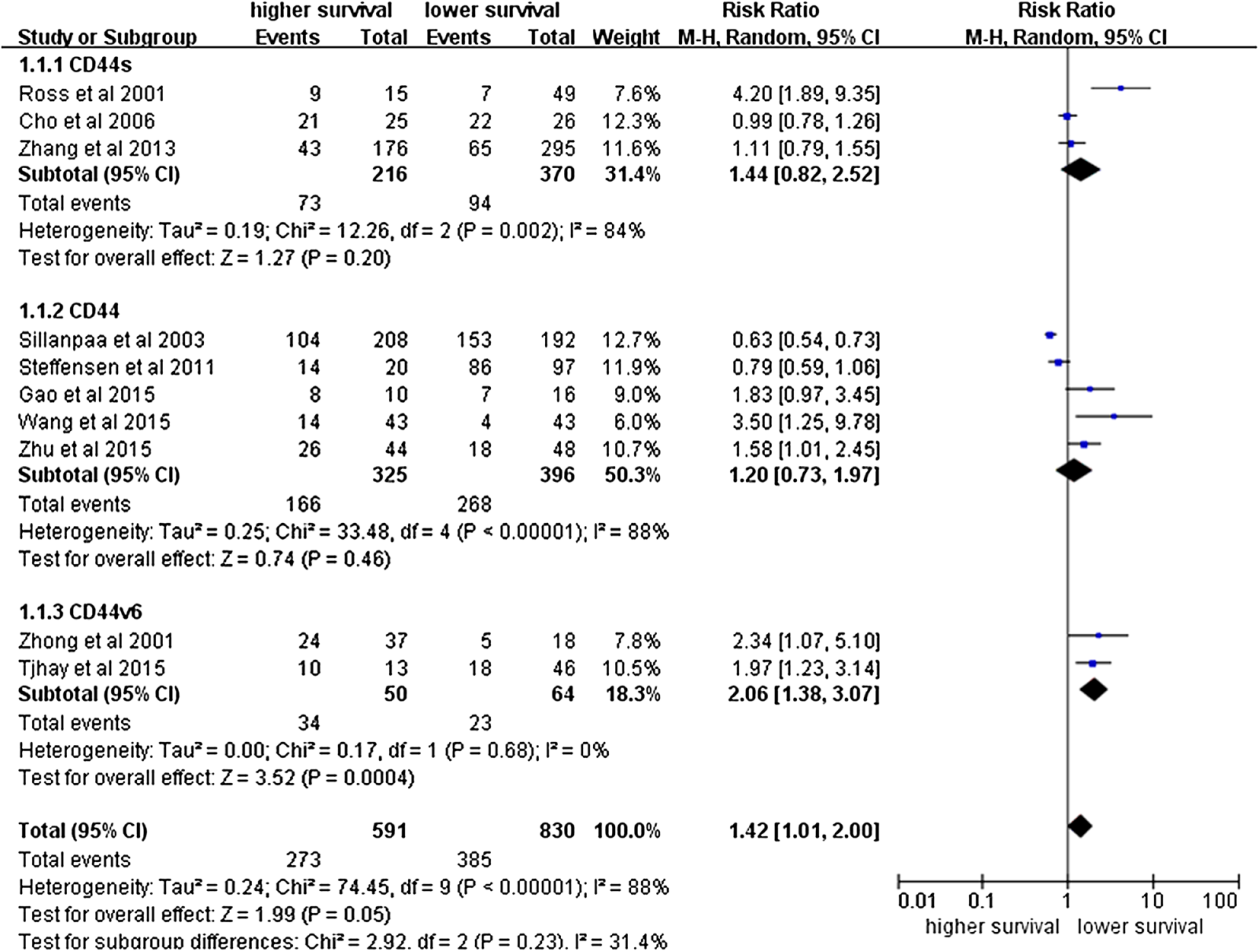
Forest plot illustrates the association between CD44 expression and OS of ovarian cancer

**Fig. 10:**
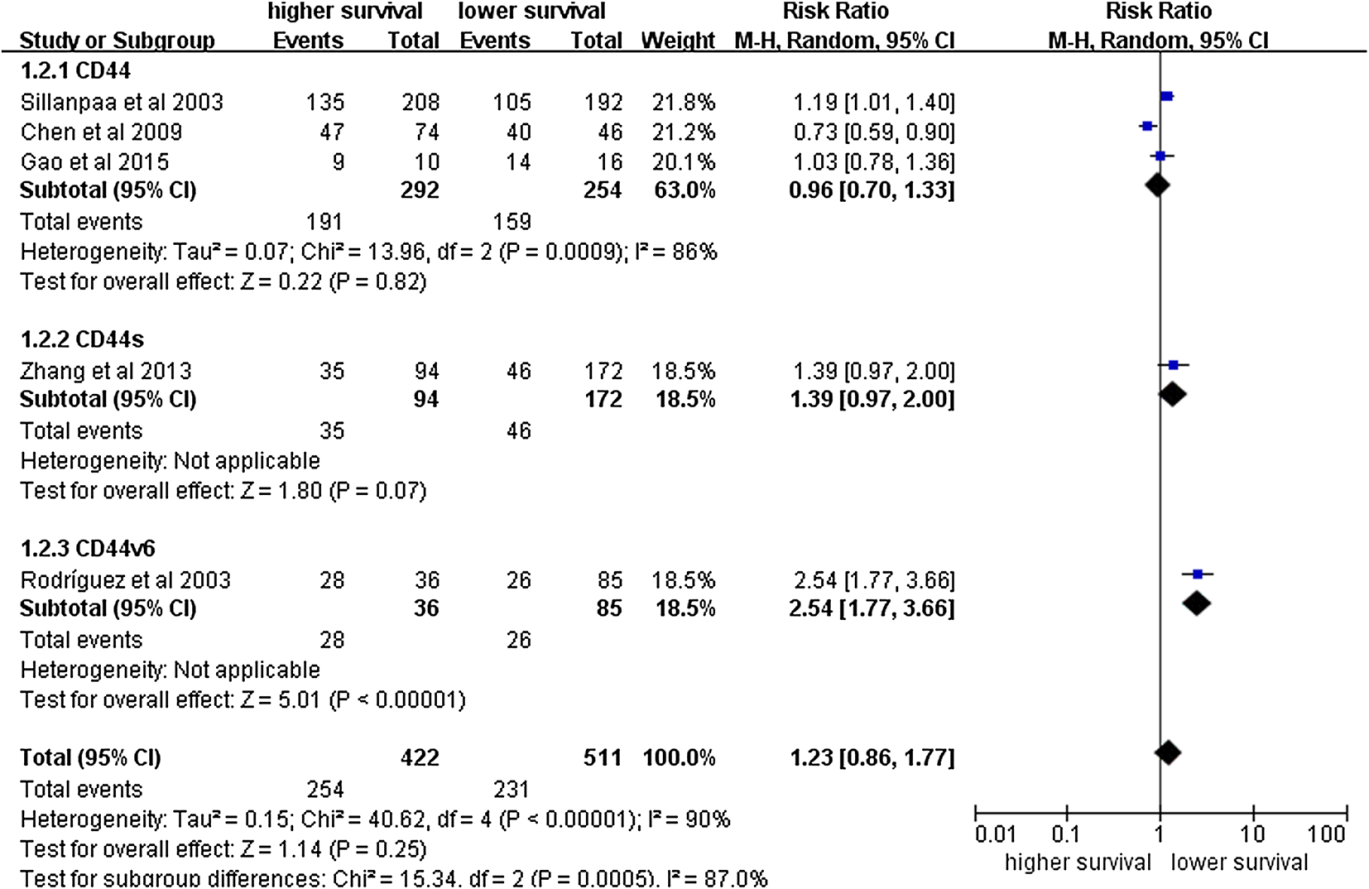
Forest plot illustrates the association between CD44 expression and DFS of ovarian cancer

Next, we analyzed the impact of CD44 expression on the survival of ovarian cancer patients. As shown in Fig. 3, our meta-analysis of the pooled data from the ten studies showed that CD44 expression is significantly associated with a poor 5-year OS (RR = 1.42, 95% CI 1.01–2.00, P = 0.05) (Fig. 9). Nevertheless, CD44 expression is not significantly correlated with DFS (RR = 1.23, 95% CI 0.86–1.77, P < 0.25) (Fig. 10).

### Sensitivity analysis

In order to rule out a bias introduced by the low numbers of eligible publications for our meta-analysis, we then performed a sensitivity analysis. For this purpose, an individual study included in the meta-analysis was removed for each round of analysis to investigate the influence of the single dataset of the particular study on the pooled ORs. Our data suggest that the corresponding pooled ORs were not significantly altered by the removal of any study (data not shown), indicating that our results are statistically robust.

### Publication bias

Next we evaluated the publication bias regarding our meta-analysis using Begg’s funnel plot and Egger’s test. The Begg’s funnel plots of the meta-analyses of the correlation between CD44 expression and the clinicopathological parameters and 5-year OS or DFS did not show an evident asymmetrical shape. Consistently, the results of Egger’s test also rule out publication bias involving our meta-analysis (Table 2).

**Table 2:** Egger’s test of funnel plot asymmetry

Clinicopathological parameters	t value	df	P value
Tumor differentiation	1.78	12	0.484
Tumor TNM stage	1.08	16	0.714
Primary residual tumor	0.66	3	0.997
Response to chemotherapy	3.42	3	0.174
Age	6.15	4	0.142
Lymphatic metastasis	2.97	5	0.243
Histology	0.66	8	0.322
Ascites	0.67	4	0.327
Overall survival	2.26	11	0.531
Disease free survival	0.21	4	1.000

*df* deflection

## Discussion

It has been hypothesized that the formation and progression of cancers are driven by CSCs which represent a minor population in cancer cells [35]. More importantly, CSCs are considered to be responsible for chemotherapy resistance, metastasis, and postoperative recurrence [36]. A significant fraction of ovarian cancer patients will ultimately develop cancer recurrence and succumb to chemo-resistant disease [37]. Furthermore, to date, there are still no reliable markers available for the diagnosis and prognosis of ovarian cancer patients. Therefore, it remains urgent to search for a reliable prognostic parameter applicable in clinical practice to predict disease outcomes in ovarian cancer. CD44 is an important cell surface marker for isolating CSCs from tumors and is correlate with poor prognosis in various human cancers [7, 38–40]. Intriguingly, in this study, we found that the expression of CD44v6 in ovarian cancers is significantly associated with a high TMN stage. Moreover, our findings revealed that CD44 expression is inversely correlated with a poor 5-year OS. However,we find no significant association between CD44 positivity and tumor grade, lymphatic metastasis, age of the patients, residual tumor size, response to chemotherapy, or ascites volume. These data indicate that CD44 expression may be used in the pathological evaluation of tissue histology to predict ovarian cancer prognosis in the future.

CD44 was identified as a surface glycoprotein and a lymphocyte homing receptor found on lymphoid and epithelial cells in 1982 [41]. Its main function on lymphocytes is mediating interaction with the endothelium [42]. Substantial evidence indicates that CD44 has been implicated in cancer invasion and metastasis. CD44v6, one of the major variants of CD44, could modulate the conjugation of CD44s and hyaluronic acid (HA), or enhance the metastasis of tumor by conjugating with HA [43]. CD44 plays an essential role in epithelial mesenchymal transition (EMT), one of the most important events in the cancer invasion process [44, 45]. Consistently, in some epithelial cells, the EMT process was accompanied by CD44 isoform switch from CD44v6 to CD44s, and CD44s has been proved to promote the EMT process [46]. Moreover, CD44 also plays a critical role in cell migration. After activated by its binding to hyaluronan, the cytoplasmic tail of CD44 in turn bind to the actin cytoskeleton and it would be translocated to the leading edge of the migrating cells. Thereafter, CD44 binds to CD62 on the endothelial cells, enabling the migrating cells to roll on the endothelia cells, which is the initial step of cell migration named extravasation [47]. Because of the important role that CD44 plays in cancer invasion and metastasis, it has been suggested that the expression of CD44 or certain CD44 variants could serve as valuable candidates for early detection, or as a prognostic predictor for gynecologic malignancies. Although some studies reported that high levels of CD44 expression was associated with a poor prognosis in ovarian cancer patients [17, 28], On the contrary, other groups concluded that CD44 expression had no influence on the survival of patients with ovarian cancer [15, 27]. This inconsistency may result from small sample size in each individual study. Here, we summarized the pooled data from these studies to increase the statistical power and better evaluate the prognostic values of the expression status of CD44 and its variants in ovarian cancer. To the best of our knowledge, this is the first meta-analysis of published data to evaluate the association between CD44 expression and prognosis in ovarian cancer.

It is important to note that there may be some potential limitations regarding our meta-analysis. First, variation in definitions of clinical outcomes, measurements and experimental procedures might contribute to between-study heterogeneity. It is particularly difficult to address the issue of variations in the definitions of clinical outcomes among different studies. Second, potential publication bias may also be a concern. We restricted our review to articles published in English or Chinese language because other languages were not accessible to the readers, which could favor the positive data that are more often published in English while the negative ones tend to be more often reported in other native languages.

## Conclusion

Our findings demonstrate that CD44 expression is associated with a higher tumor TNM stage among ovarian cancer patients. Moreover, ovarian cancer patients with positive CD44 expression exhibit a worse clinical outcome than those with negative CD44 expression. Further studies with larger sample sizes will be warranted to validate the findings of our meta-analysis in the future.

## Abbreviations

IHC: immunohistochemistry
OS: overall survival
DFS: disease-free survival
ND: not document
